# Single-Cell Transcriptome Analysis Dissects the Replicating Process of Pancreatic Beta Cells in Partial Pancreatectomy Model

**DOI:** 10.1016/j.isci.2020.101774

**Published:** 2020-11-06

**Authors:** Hisato Tatsuoka, Satoko Sakamoto, Daisuke Yabe, Ryotaro Kabai, Unyanee Kato, Tatsuya Okumura, Ainur Botagarova, Shinsuke Tokumoto, Ryota Usui, Masahito Ogura, Kazuaki Nagashima, Eri Mukai, Yoshio Fujitani, Akira Watanabe, Nobuya Inagaki

**Affiliations:** 1 Department of Diabetes, Endocrinology and Nutrition, Kyoto University Graduate School of Medicine, Kyoto, Japan; 2 Medical Innovation Center, Kyoto University Graduate School of Medicine, Kyoto, Japan; 3 Department of Diabetes and Endocrinology, Gifu University Graduate School of Medicine, Gifu, Japan; 4 Yutaka Seino Distinguished Center for Diabetes Research, Kansai Electric Power Medical Research Institute, Hyogo, Japan; 5 Division of Molecular and Metabolic Medicine, Department of Physiology and Cell Biology, Kobe University Graduate School of Medicine, Hyogo, Japan; 6 Laboratory of Medical Physiology and Metabolism, Department of Biomedical Sciences, College of Life Sciences, Ritsumeikan University, Shiga, Japan; 7 Laboratory of Developmental Biology and Metabolism, Institute for Molecular & Cellular Regulation, Gunma University, Gunma, Japan

**Keywords:** Biological Sciences, Molecular Biology, Genomics

## Abstract

Heterogeneity of gene expression and rarity of replication hamper molecular analysis of β-cell mass restoration in adult pancreas. Here, we show transcriptional dynamics in β-cell replication process by single-cell RNA sequencing of murine pancreas with or without partial pancreatectomy. We observed heterogeneity of *Ins1*-expressing β-cells and identified the one cluster as replicating β-cells with high expression of cell proliferation markers *Pcna* and *Mki67*. We also recapitulated cell cycle transition accompanied with switching expression of *cyclins* and E2F transcription factors. Both transient activation of endoplasmic reticulum stress responders like *Atf6* and *Hspa5* and elevated expression of tumor suppressors like *Trp53*, *Rb1*, *and Brca1* and DNA damage responders like *Atm*, *Atr*, *Rad51*, *Chek1*, and *Chek2* during the transition to replication associated fine balance of cell cycle progression and protection from DNA damage. Taken together, these results provide a high-resolution map depicting a sophisticated genetic circuit for replication of the β-cells.

## Highlights

• Single cell RNA sequencing dissects a sequence of replication process of beta cells
• ER stress responders are transiently activated in initiation of the proliferation
• Physiological replication accompanied with induced expression of tumor suppressors
• Fine balance of proliferation genes and tumor suppressors is a key of the replication

## Graphical Abstract

**Figure:**
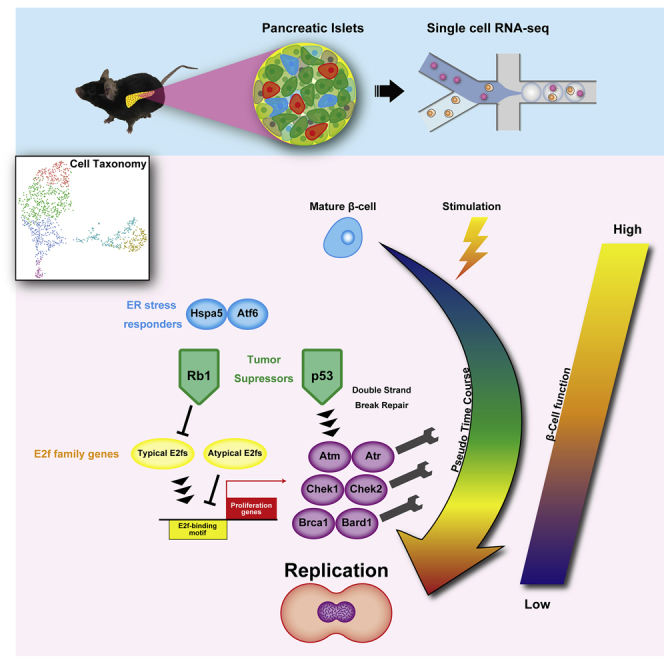

## Introduction

Type 2 diabetes is characterized by impaired insulin secretion resulting from dysfunction of the pancreatic β-cells and/or reduction in β-cell mass in addition to insulin resistance in peripheral tissues (Fujimoto and Inagaki, 2011; Kahn, 2003; Yabe et al., 2015). Current anti-diabetes drugs targeting β-cell dysfunction enhance insulin release from β-cells (Seino et al., 2017) but exhibit limited effect to patients whose β-cell mass is already reduced. Since developing new drugs for the patient with low β-cell mass has been highly desired, several strategies for increment of intrinsic β-cell mass are proposed, such as stimulating β-cell replication, decreasing β-cell death, and producing mature β-cells from progenitors or other types of cells (Karaca et al., 2009). Previous studies showed self-replication of pre-existing β-cells plays the most important role in the maintenance of β-cell mass in adult mice (Dor et al., 2004; Teta et al., 2007). However, replication rate of β-cells in adult human and mice is extremely low (Butler et al., 2003; Reers et al., 2009; Teta et al., 2005) as a result of its gradual decrease with aging from highest replication after birth (Gregg et al., 2012; Teta et al., 2005). Thus, an attempt to increase β-cell mass by stimulating the replication is a major challenge in the treatment of diabetes, and the mechanisms involved in β-cell replication in adult are eagerly sought.

To study molecular mechanisms of β-cell replication in adult pancreas, several animal models have been developed: acquisition of insulin resistance (El Ouaamari et al., 2016; Terauchi et al., 2007), induction of hyperglycemia (Sharma et al., 2015), pregnancy model (Kim et al., 2010), β-cell ablation (Morita et al., 2016), administration of a glucokinase activator (Nakamura et al., 2009), and partial pancreatectomy (PPTx) model (Peshavaria et al., 2006; Rankin and Kushner, 2009; Togashi et al., 2014). Among these efforts, PPTx has been shown to offer the most robust replication of murine β-cells without critical effect on blood glucose. A microarray analysis shows altered expression of cell cycle genes and the *Reg* gene family by PPTx but could not address whether the alterations recapitulated replicating β-cell-specific gene networks (Rankin and Kushner, 2010), probably due to the fact that islets are composed of multiple cell types such as α-, δ-, PP, and β-cells including a small number of replicating cells (Bader et al., 2016; Dorrell et al., 2016; Johnston et al., 2016; Steiner et al., 2010). Moreover, and more importantly, understanding of similarity or difference between physiological proliferation and malignant tumorigenesis is required for secure induction of replication of functional β-cells. Molecular understanding for fine control of the proliferation, a double-edged sword, with a brake against aberrant growth leads to development of safe pharmaceuticals (Heit et al., 2006). However, physiological condition-specific proliferation signaling has not been clarified.

Single-cell RNA sequencing (scRNA-seq) has enabled to highlight β-cell subpopulations based on their gene expression profiles in human and mice (Baron et al., 2016; Muraro et al., 2016; Segerstolpe et al., 2016) and describe gradual change of transcriptome by pseudo-time course analysis (Xin et al., 2018; Zeng et al., 2017). However, dynamics of gene expression in the transition to the proliferating state remains unknown. In the current study, we employed scRNA-seq to delineate taxonomy of islet cells of young mice and addressed molecular circuit activating replication of the β-cells.

## Results

### Alteration of Gene Expression in PPTx-Induced β-Cell Proliferation

**Figure 1:**
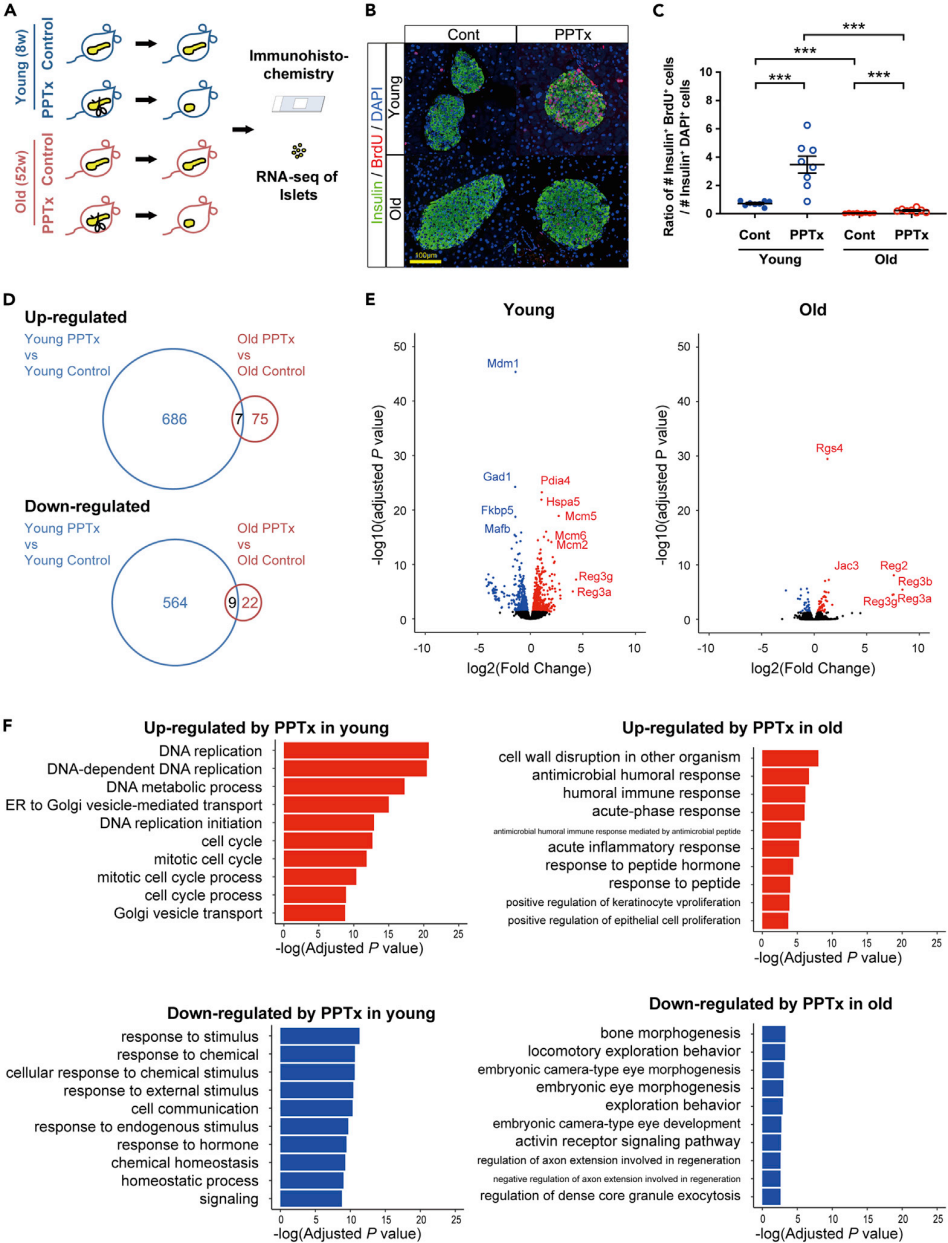
Altered Gene Expression Profile of Pancreatic β-cells after Partial Pancreatectomy (PPTx) (A) Experimental design for analyses of replication of β-cells using PPTx mice. Young and old C57BL/6J mice were subjected to PPTx followed by immunohistochemistry and RNA sequencing. The control group received no surgical operation. (B) Representative image of pancreatic β-cells labeled with 5-bromo-2′-deoxyuridine (BrdU). Scale bar represnts 100 μm. The pancreas from young and old mice in the control and PPTx groups were immunostained for insulin (green), BrdU (red), and 4′,6-diamidino-2-phenylindole (DAPI) (blue). (C) Ratio of insulin^+^BrdU^+^ cells to insulin^+^DAPI^+^ cells. Values are mean ± SEM. ∗∗∗, p < 0.001 (Mann-Whiteny U test). (D) Numbers of differentially expressed genes in bulk RNA sequencing analysis between islets of control and 2 days after PPTx in young (blue) and old (red) mice (n = 4, each group). Numbers of upregulated genes (upper panel) and downregulated genes (lower panel) in the PPTx group compared to control ones were shown. (E) Volcano plots of differentially expressed genes in bulk RNA sequencing of the control and PPTx group in young (left) and old mice (right) (n = 4, each group). Genes significantly upregulated and downregulated in PPTx groups are indicated in red and blue, respectively. Adjusted p value <0.05 was defined as significant. (F) Gene ontology (GO) terms enriched in genes upregulated (upper panel) or downregulated (lower panel) in the PPTx group compared to control in young (left) and old (right) mice. The top 10 GO terms are shown for each comparison. See also Figure S1 and Table S1.

To activate replication of β-cells, we conducted 50% PPTx on 8-week-old (young) and 52-week-old (old) C57BL/6J mice as described previously (Peshavaria et al., 2006) (Figure 1A) and observed that PPTx induced mild hyperglycemia both in young and old mice, whereas obvious changes were not seen in body weight, glucose tolerance, and insulin secretion (Figures S1A–S1D), in agreement with previous reports (Rankin and Kushner, 2009; Togashi et al., 2014). *In vivo* labeling of islet cells with 5-bromo-2′-deoxyuridine (BrdU) demonstrated that proliferating insulin^+^/BrdU^+^ cells were significantly increased by PPTx, and increment of the cells in young mice which underwent PPTx was much greater than that in old mice (Figures 1B and 1C), which is also in agreement with a previous study (Rankin and Kushner, 2009).

We then conducted bulk RNA-seq of PPTx and control islets in young and old mice (Figures 1D and S1E) and observed induction of *Reg3a* and *Reg3g* by PPTx both in young and old mice as reported previously (Figure 1E) (Rankin and Kushner, 2010). In addition, we found young PPTx-specific induction of a large number of genes including a well-known endoplasmic reticulum (ER) marker *Hspa5* (GRP78) and DNA replication licensing factors *Mcm2*, *Mcm5*, and *Mcm6* (Figure 1E). Gene ontology (GO) enrichment analysis also showed that genes related to cell cycle and ER function were remarkably upregulated in young mice which underwent PPTx compared to its control, whereas high expression of inflammation and immune response genes was observed in old mice which underwent PPTx (Figure 1F; Table S1).

### Cell Taxonomy by Single-Cell Transcriptome Profiling Highlights Replicating β-Cells

**Figure 2:**
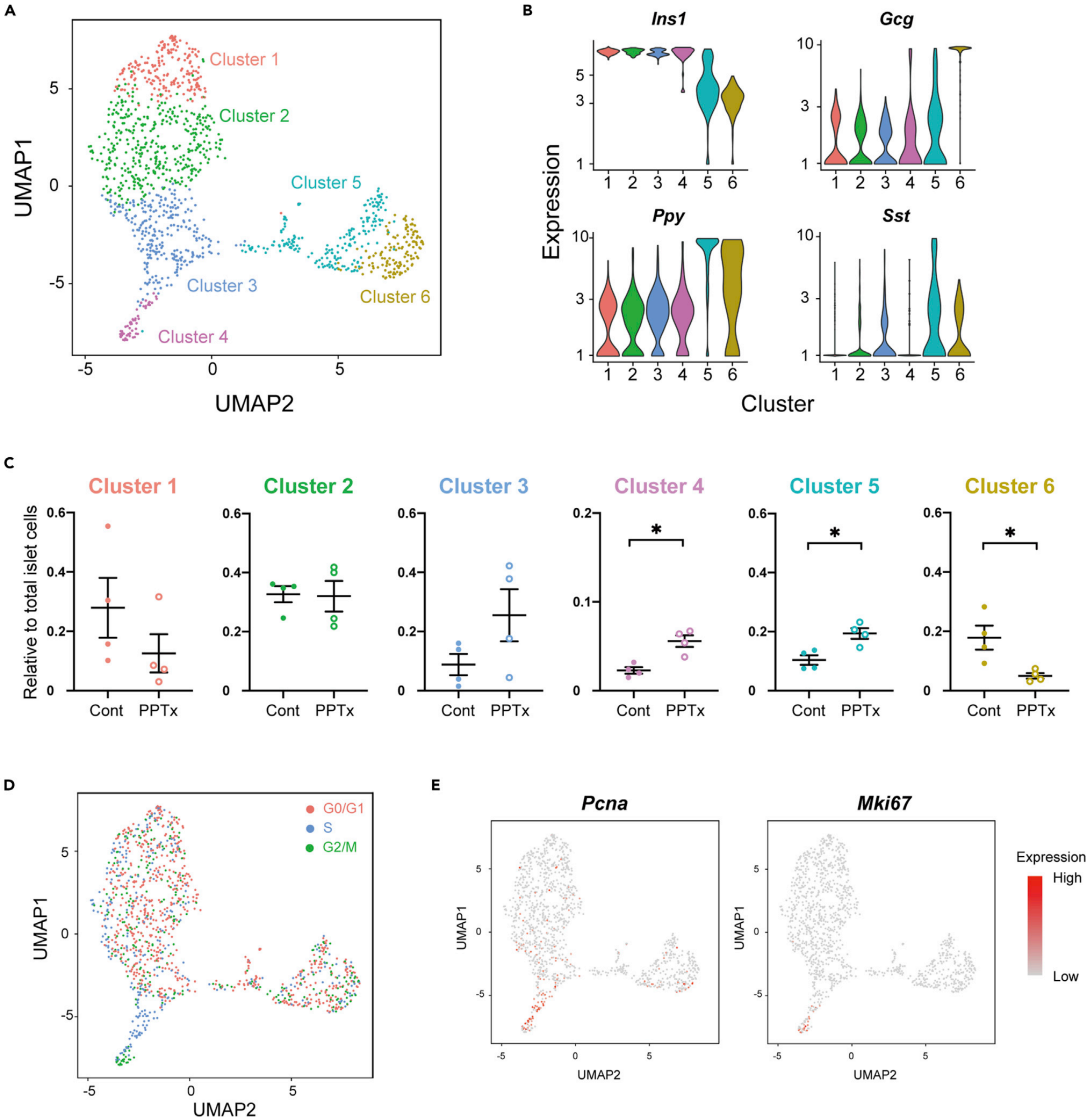
Cell Taxonomy and Pseudo-Time Course Analysis by Single-Cell Transcriptome Profiling of Islet Cells (A) Uniform manifold approximation and projection (UMAP) plot of pancreatic islet cells from PPTx and control mice (PPTx, 712 cells from 4 samples; control, 520 cells from 4 samples). (B) Gene expressions of endocrine hormones (*Ins1*, *Gcg*, *Ppy*, and *Sst*) in each cluster. (C) Relative proportions of cell numbers in each cluster to total cell numbers. Values are mean ± SEM. ∗, p < 0.05 vs control (Mann-Whitney U test). (D) Estimation of cell cycle in each cluster. Red color, G0/G1 phase; blue, S phase; green, G2/M. (E) The expressions of *Pcna* and *Mki67* on UMAP plot.

Our conventional bulk RNA-seq could not state whether altered gene expression profiles represented the majority of islet cells or reflected strong induction of the genes in a rare population such as replicating β-cells. Thus, we conducted scRNA-seq of PPTx and control islet cells from young mice to highlight gene expression signatures of replicating β-cells. Dimensionally, reduction by uniform manifold approximation and projection (UMAP) classified the islet cells into 6 subpopulations (Figure 2A), and we defined each cluster as β-cells (*Ins1*-expressing clusters 1–4), PP- and δ-cells (*Ppy*- or *Sst*-expressing cluster 5), and α-cells (*Gcg*-expressing cluster 6) (Figures 2B and S2A). In the clusters with *Ins1* expression, a proportion of cells in the cluster 4 exhibited statistically significant difference between PPTx and control groups (Figure 2C), proposing this cluster was a replicating β-cell population. A proportion of cluster 4 cells was concordance with immunohistochemistry of BrdU^+^ cells (BrdU^+^, 0.73% in control vs 3.47% in PPTx; and cluster 4 cells, 2.3% in control vs 5.6% in PPTx) and exhibited high reproducibility in replicates (N = 4 in Figures 1C and 2C). Cell cycle scoring analysis also demonstrated that all cluster 4 cells were in S and G2/M phase, whereas the other β-cell clusters exhibited mixed status of cell cycle (Figures 2D and S2B; Table S2). We further confirmed high expression of cell-cycle-related genes including *Mki67* (Ki67) and *Pcna* (Figures 2E and S2C; Table S3) and concluded that cluster 4 cells were replicating β-cells. While GO analyses also showed differential expression of cell-cycle-related genes in cluster 4 cells, dominant signatures of differentially expressed genes were downregulation of protein transport pathways in cluster 1 and upregulation of ER stress responders, such as *Hsp90b1*, *Hspa5*, *Ssr3*, and *Manf*, in cluster 3 (Figure S2C; Table S3).

### Activation of ER Stress and DNA Damage Responders during Transition to Proliferative State of β-Cells

**Figure 3:**
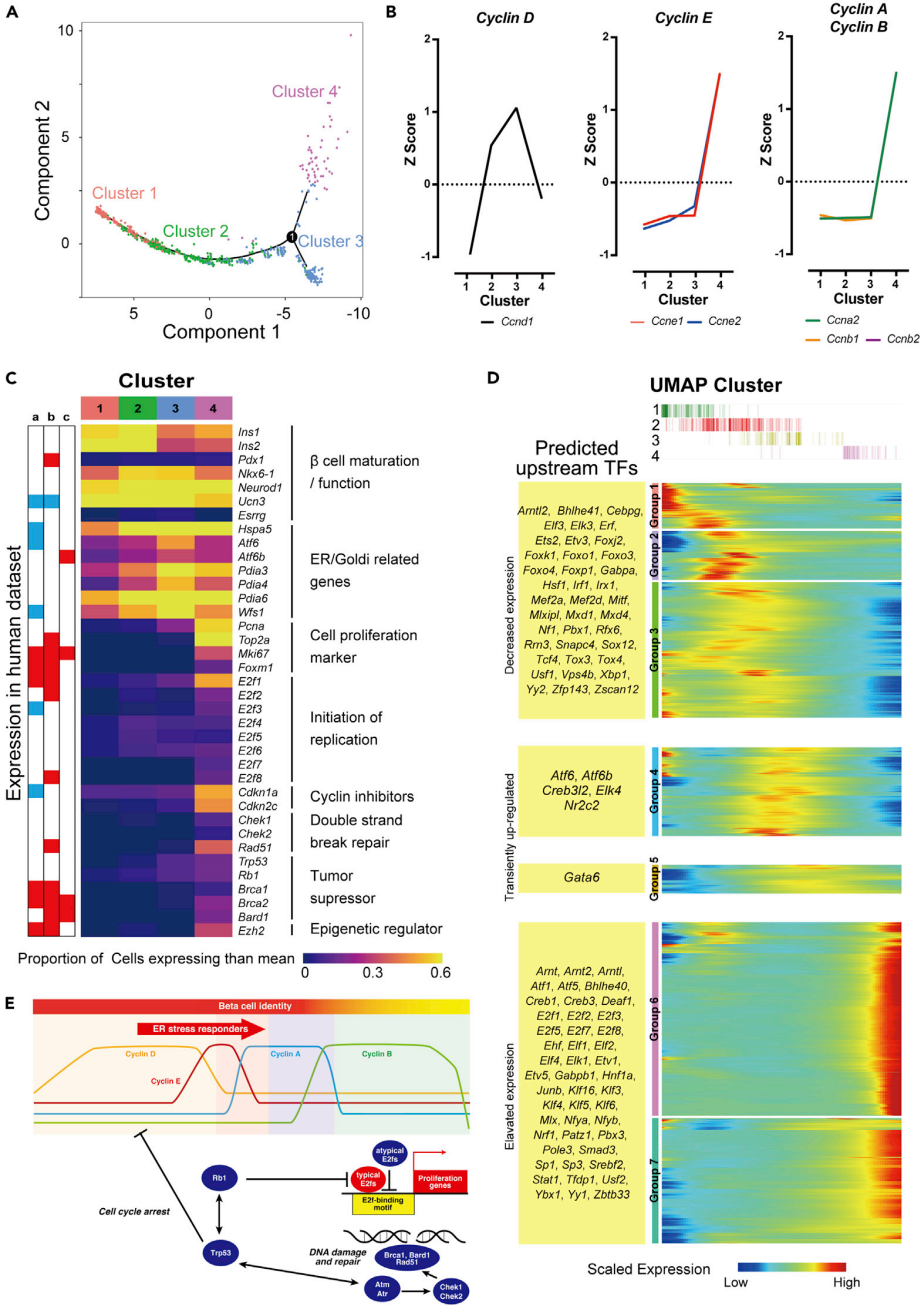
Dynamics of Gene Expression and Regulation in a Transition to Replication of β-cells (A) Trajectory plot showing transition of cells from the cluster 1 through the cluster 2 and 3 to the cluster 4. (B) Dynamic expression of cell-cycle-specific cyclins in the UMAP clusters. The *Z* score of mean expression in each cluster was shown. (C) The expression score of genes related to β-cell maturation and function, ER stress, cell proliferation, initiation of replication, cyclin inhibitors, double-strand break repair, tumor suppressor, and epigenetic regulator in Ins1^+^ cluster on UMAP (i.e., cluster 1–4). Scores were calculated with the number of cells which expression values of the gene were more than its mean expression among cluster 1–4 cells divided by total cell numbers of individual clusters. Row annotation located on the left shows the association to genes reported in previous studies as for human β-cell: (A) human insulinoma (Wang H, et al., 2017), (B) replication-stimulated human islets by Dyrk1a inhibitor (Ackeifi et al., 2020), and (C) juvenile human islets (Arda et al., 2016). Red, upregulated genes; blue, downregulated in proliferative tissues or cells. (D) Dynamics of gene regulations were depicted using the pseudo-temporal expression pattern, showing a rough fit to a trajectory of the UMAP clusters. The pattern of gene expression in pseudo-time was classified into 7 groups (group 1–7). Candidate upstream regulators whose expression exhibited the same pattern of gene expression were listed on the left. (E) Schematic of a sequence of replication process of β-cell replication with switching gene regulation. Switching expression of *cyclins* and transient activation of ER stress responders is followed by induction of a series of tumor suppressors and DNA damage responders002C as well as high expression of cell proliferation-related genes. See also Figure S3 and Tables S6 and S7.

Pseudo-time course analysis displayed a trajectory of the cells from cluster 1 through clusters 2 and 3 to cluster 4 (Figure 3A), demonstrating that the trajectory recapitulated a process of transition to the replicating state of the β-cells. We indeed observed switching expression of *cyclins* in a trajectory recapitulated cell cycle transition from G0/G1, S to G2/M phase (Figure 3B; Table 1): upregulation of late G1 genes *Ccnd1* from cluster 1 to cluster 2; upregulation of *Ccne2* (G1-to-S) from cluster 2 to cluster 3; bursting expression of *Ccne1*, *Ccne2*, and *Cdk2* (G1-to-S), *Ccna2* (S), and *Ccnb1*, *Ccnb2*, and *Cdk1* (G2) from cluster 3 to cluster 4. We next addressed gene networks responsible for replication of the β-cells. Detailed look of the differentially expressed genes using the ingenuity pathway analysis and GO suggested a sequential alteration of gene expression in a transition to replication of the β-cells (Figures 3C and S3A; Tables 1 and S4). ER/Golgi-related genes including *Atf6*, *Hspa5*, *Wfs1*, and *Pdia* family and a set of tumor suppressor genes *Trp53* (murine p53), *Rb1*, and *Brca1* were activated at clusters 2 and 3 (Figure 3C; Table 1). Then, expressions of E2F family genes such as *E2f1*, *E2f2*, and *E2f3*, cell proliferation markers including *Top2a*, *Pcna*, *Foxm1*, and *Mki67*, cyclin inhibitors *Cdkn1a* (p21), *Cdkn2a* (p14^ARF^/p16^INK4A^), and *Cdkn2c* (p18), and regulators of a double-strand break repair such as *Chek1*, *Chek2*, and *Rad51* were elevated to cluster 4 (Table 1). We also observed upregulation of epigenetic regulators like *Dnmt3a*, *Mecp2*, *Ezh2*, and *Hells* in cluster 4 (Table 1).

**Table 1:** List of Representative Genes Differentially Expressed between the Two Clusters

	Cluster 1vs 2	Cluster 2 vs 3	Cluster 3 vs 4
Cell Cycle			

*Ccnd1*^a^	1.44	NA	NA
*Ccne1*	NA	NA	17.00
*Ccne2*	NA	2.45	6.37
*Ccna2*^a^	NA	NA	125.47
*Ccnb1*^a^	NA	NA	69.81
*Ccnb2*^b^	NA	NA	111.28
*Cdk1*^b^	NA	NA	107.92
*Cdk2*	NA	NA	11.08
*Cdk4*	0.99	NA	1.82
*E2f1*^a^^,^^b^	NA	1.26	5.48
*E2f2*^b^	7.38	0.17	20.81
*E2f3*	NA	NA	9.46
*E2f4*	1.81	NA	NA
*E2f7*	NA	NA	27.45
*E2f8*^b^	NA	NA	
*Mki67*^a^^,^^b^^,^^c^	NA	NA	98.93
*Top2a*^b^	NA	NA	171.52
*Pcna*	NA	4.54	6.36
*Foxm1*^a^^,^^b^	NA	NA	96.66

ER Stress			

*Atf6*	NA	1.57	0.62
*Atf6b*^c^	1.03	1.26	NA
*Hspa5*	1.45	1.57	0.73
*Pdia3*	1.05	1.76	NA
*Pdia4*	2.03	1.88	0.57
*Pdia6*	1.41	1.42	0.86
*Wfs1*	1.13	1.27	0.63
*Hells*^d^^,^^a^^,^^b^	NA	3.76	7.86

Tumor Suppressor/DNA Damage			

*Trp53*	NA	3.77	NA
*Cdkn1a*	NA	NA	5.64
*Cdkn2a*	NA	NA	
*Cdkn2c*	NA	2.27	15.67
*Rb1*	NA	1.47	NA
*Brca1*^a^^,^^b^	NA	∞	4.35
*Brca2*^a^^,^^b^^,^^c^	NA	∞	20.19
*Bard1*^b^^,^^c^	NA	NA	76.70
*Atr*	NA	2.38	NA
*Atm*	1.77	NA	2.44
*Rad51*^b^	NA	NA	23.30
*Chek1*	NA	NA	9.63
*Chek2*	NA	NA	

Epigenetic Regulators			

*Dnmt3a*	NA	1.42	0.49
*Mecp2*	NA	1.32	NA
*Ezh2*^a^^,^^b^	∞	NA	95.95

Fold change is calculated by dividing the cpm value of the latter cluster by that of the former cluster. NA, no statistical significance between clusters; ∞, undefined high expression because expression of the denominator gene was zero.

a Also included in upregulated genes in the insulinoma data set (Wang et al., 2017).

b Also included in upregulated genes in the human islets treated by harmine (Ackeifi et al., 2020).

c Also included in upregulated genes in juvenile human islets (Arda et al., 2016).

d Also included in Epigenetic regulators.

To address whether the transcriptional signature of β-cell replication observed in mice is modeled on replication process of human pancreatic β-cells, we compared to human insulinoma (Wang et al., 2017), Dyrk1a inhibitor-induced β-cell replication (Ackeifi et al., 2020), and juvenile human islets (Arda et al., 2016). Of 440 genes enriched in cluster 4, 68, 108, and 19 genes were overlapped, respectively, and among them, the overlapped genes included replication-related genes such as *Mki67*, *Foxm1*, *Ccna2*, *Ccnb1*, *Ccnb2*, and *Ccne2* and tumor suppressor genes *Brca1*, *Brca2*, or *Bard1* (Table S5). In addition, E2f family genes of *E2f1*, *E2f2*, or *E2f8* and epigenetic regulator *Ezh2* or *Hells* were also upregulated in human data sets (Figure 3C; Table 1).

Dedifferentiation is a process of loss of specialized function, but occasionally, dedifferentiation accompanied with acquisition of different features: one is transdifferentiation to give functional somatic cells and another is reversion of differentiation process, which gives progenitor cells that can redifferentiate into several cell types. Expressions of genes responsible for β-cell function such as *Ins1*, *Ins2*, *Pdx1*, *Nkx6*.*1*, *Neurod1*, *Ucn3*, and *Esrrg* were declined toward cluster 4 (Figures 3C, S2C, and S3A; Table S3), indicating dedifferentiation occurs in replicating cells, in terms of loss of function (Puri et al., 2018). We then examined a possibility of transdifferentiation or reversion to progenitors. The replicating cells did not exhibit expressions of markers of immature and/or progenitor cells such as *Neurog3* or *Oct4* and *Nanog* (Talchai et al., 2012). And expression levels of the other endocrine cells like *Gcg*, *Ppy*, or *Sst* were not altered. These observations explain that our replicating cells simply lose a function as β-cells but do not exhibit any evidence of transdifferentiation and a reversion of differentiation. Taken together, replication of the β-cell is a combined process of suppression of cell identity, activation of ER stress responders, and simultaneous upregulation of tumor suppressor genes toward cell cycle progression under surveillance of DNA damage.

We finally examined molecular mechanism of transcriptional regulation raising dynamic changes in gene expression during the transition. We examined motifs enriched on promoters of differentially expressed genes and observed high enrichment of E2F-binding motifs on promoters of cluster 4 genes (Figure S3B). While DNA-binding motif among E2F family proteins is very similar and indistinguishable, gene expression profile suggested that *E2f1*, *E2f2*, *E2f3*, *E2f7*, and *E2f8*, which were upregulated in cluster 4, were candidate regulators of replication-related genes through their binding motifs (Figure 3C). A further analysis of transiently expressed genes demonstrated Atf6/Atf6b-binding motifs on promoters of ER stress-related genes including *Atf6* and *Atf6b* themselves (Figure 3D; Tables 1, S6, and S7).

## Discussion

It has been challenging to unravel the molecular mechanisms regulating β-cell replication due to the rarity of replicating β-cells and the heterogeneity of islet cells (Teta et al., 2005). Our high-resolution map of transcriptional dynamics in transition to the replication shows switching expression of *cyclins* and identifies proliferating β-cells. We show that ER stress responders, which were reported to be involved in proliferation of murine β-cells (Sharma et al., 2015; Usui et al., 2012), expressed transiently and prior to *cyclin E*, *cyclin A*, and *cyclin B*, indicating an importance of fine protein folding and quality control during cell cycle progression. The *Wfs1*, a component of unfolded protein response complex, was transiently activated, as well as the other ER stress responders. Genomic defect of the *Wfs1* gene causes Wolfram syndrome through loss of human β-cells (Hofmann et al., 2003; Ishihara et al., 2004), supporting an importance of appropriate proteostasis in the β-cells. Notably, expression of the ER stress responders was induced by PPTx only in young mice but not in old mice (Figure S3C), concordant with low potency of murine β-cell replication with age (Aguayo-Mazzucato et al., 2017; Krishnamurthy et al., 2006; Rankin and Kushner, 2010). Dysregulation of ER stress-related genes in aged mice and diseases associates new strategies to explore drug target genes by understanding the transcriptional dynamics of β-cell replication (Naidoo et al., 2014). We show not only heterogeneous expression of ER stress-related genes in pancreatic human and murine β-cells (Baron et al., 2016; Muraro et al., 2016; Xin et al., 2018) but also sophisticated connection of ER stress signaling to the other gene networks like cell cycle regulators and DNA damage responders.

We observed complicated regulation of E2f signaling. High expression of typical E2fs, such as *E2f1*, *E2f2*, and *E2f3* (Figures 3C and Table 1), and downstream genes of the *E2fs* implied that replication of β-cells was driven by these E2f signaling (Figure S3B; Tables S4 and S7), as shown in previous reports (Fajas et al., 2004; Hanahan and Weinberg, 2000; Iglesias-Ara et al., 2015; Rady et al., 2013; Thurlings and de Bruin, 2016; Wong et al., 2011). However, we also observed that *Rb1*, a cell cycle inhibitor through binding to E2Fs, expressed prior to upregulation of *E2fs* (Table S4). Poised expression of *Rb1* for control of typical *E2fs* may contribute to adequate proliferation of the β-cells. In addition, we also observed high expression of atypical E2F family genes *E2f7* and *E2f8* (Figure 3C), which repress expression of replication-related genes as a member of p53 signaling (Carvajal et al., 2012). Interestingly, expressions of *E2F7* and *E2F8* were also induced by Dyrk1a inhibitor that promotes replication of human β-cells (Wang et al., 2015). We have recently reported that *Rb*-deficient mice with mutant *Trp53* develop aggressive insulin-secreting tumors and die by the age of 10 months (Yamauchi et al., 2020). This indicates synergistic roles of the *Rb1* with *Trp53* in tumor suppression of the pancreatic β-cells. Bidirectional regulation of E2f signaling that is involved in both promotion and suppression of cell cycle is a paradox to be unraveled.

We observed the induction of *Trp53* expression in cluster 3, followed by high induction of p53-target genes including *Chek1* and *Chek2* in cluster 4, and also found that *Atm*, *Atr*, *Brca1*, *Brca2*, *Hells*, *Bard1*, and *Rad51*, which maintain genome integrity in response to DNA damage, were induced during the transition (Tables 1 and S4). Activation of the tumor suppressors prior to cell cycle progression may also be a gatekeeper of precise cell cycle regulation in physiological replication to avoid tumorigenicity (Figure 3E; Tables 1 and S4). We additionally observed alteration of expression of epigenetic regulators like *Dnmt3a*, *Mecp2*, *Hells*, and *Ezh2* in transition during replication process (Table 1). A previous study reported dysregulation of epigenetic factors including genomic amplification of *EZH2* and overexpression of *EZH2* promoted *CCND1*-induced proliferation of insulinoma cells in human (Wang et al., 2017), whereas declined functions of these factors with aging were previously reported in mice (Chen et al., 2009). Thus, molecular understandings of gene regulation of the epigenetic factors in β-cell replication are further challenges to complete the overall picture.

Comparing control- and PPTx-derived cells, several differences were observed, including a higher induction of ER stress-related genes in PPTx-derived cells in clusters 1, 2, 3, and 4 (Figure S3A) and a biased distribution of control- and PPTx-derived cells in cluster 4 (Table S2). Although we confirmed key findings including elevated expression of proliferation-related genes and tumor suppressors both in PPTx and control mice, PPTx might activate replication inducibly through ER stress. From another perspective, we provide transcriptional signature of physiological replication of β-cells, which was obtained from control mice without any perturbations, such as any surgical operations and genetic modification (Figure S3A). Alteration of this physiological replication in aging is the next crucial issue to be examined.

We have also considered whether the replication is driven in a subpopulation of mature β-cells with proliferation capability or follows a “stochastic” model, which says that all most of β-cells harbor potency to replicate and several cells stochastically enter in S and G2/M phases. We cannot exclude any possibility of existence of “elite” cells in clusters 1–3, although our pseudo-temporal analysis suggested transition of cells from cluster 1 to cluster 4 via clusters 2 and 3 (Figures 3A and 3D). Additional question remained is why old β-cells lose the capacity to replicate. The old mice which we employed correspond to middle-aged human, whose β-cells also lose the capacity to replicate (Dutta and Sengupta, 2016). We observed differential regulation of ER stress responders between young and old mice (Figure S3C). Since middle-age-onset diabetes is also clinically important, further study using scRNA-seq or epigenetic examination is needed (Zou et al., 2016). A map of β-cell replication process altered in aging could also be a guide for cure of diabetes.

In summary, our scRNA-seq of replication-induced islet cells addresses transcriptional dynamics of β-cell replication process at high resolution, including switching expression of cyclins, transient activation of ER stress responders, and elevated expression of proliferation-related genes. Additionally, the study provides new findings including activation of anti-proliferation signaling, such as key factors responsible for surveillance of genome integrity, orchestrated by major tumor suppressor genes. Our integrated concept for physiological induction of beta cells by both acceleration and suppression of cell proliferation will link to new strategy for regenerative medicine and drug discovery.

### Limitations of the Study

In this study, we employed the PPTx model to clarify β-cell replication. The phenomena observed in this study might not apply in other models. Our research was mostly observational; the mechanism of β-cell replication requires further investigation.

### Resource Availability

#### Lead Contact

Further information and requests for resources and reagents should be directed to and will be fulfilled by the Lead Contact, Nobuya Inagaki (inagaki@kuhp.kyoto-u.ac.jp).

## Materials Availability

This study did not generate new unique reagents.

### Data and Code Availability

The data sets/code generated during this study are available at NCBI Gene Expression Omnibus (GEO) accession number: GSE152730 and GSE152731.

## Methods

All methods can be found in the accompanying Transparent Methods supplemental file.

## Supplemental Information

Document S1. Transparent Methods, Figures S1–S3, and Table S2

Table S1. Differentially Expressed Genes (DEGs) between Control and PPTx Mice both in Young and Old Mice, Related to Figure 1

Table S3. Differentially Expressed Genes (DEGs) in the Target Cluster, Related to Figure 2

Table S4. List of Pathways Associated with Cluster Specific Genes, Related to Figure S3 and Table 1

Table S5. Overlap between Human Dataset vs Genes Enriched in our Cluster 4, Related to Figure 3C

Table S6. Top 10 Gene Ontology Terms of Genes in Each Group Related to Pseudo-Time, Related to Figure 3D

Table S7. Motif Analysis for Genes Showing Pseudo-Temporal Expression Pattern, Related to Figure 3D
