# Supplementary material for: Single-Cell Transcriptome Analysis Dissects the Replicating Process of Pancreatic Beta Cells in Partial Pancreatectomy Model
Source: iScience. 2020 Nov 6;23(12):101774. doi: 10.1016/j.isci.2020.101774 (PMC7689163; doi:10.1016/j.isci.2020.101774)
Supplement: Document S1. Transparent Methods, Figures S1–S3, and Table S2 [file mmc1.pdf]

iScience, Volume *23*

Supplemental Information

Single-Cell Transcriptome Analysis Dissects the Replicating Process of Pancreatic Beta Cells in Partial Pancreatectomy Model

Hisato Tatsuoka, Satoko Sakamoto, Daisuke Yabe, Ryotaro Kabai, Unyanee Kato, Tatsuya Okumura, Ainur Botagarova, Shinsuke Tokumoto, Ryota Usui, Masahito Ogura, Kazuaki Nagashima, Eri Mukai, Yoshio Fujitani, Akira Watanabe, and Nobuya Inagaki

Figure S1

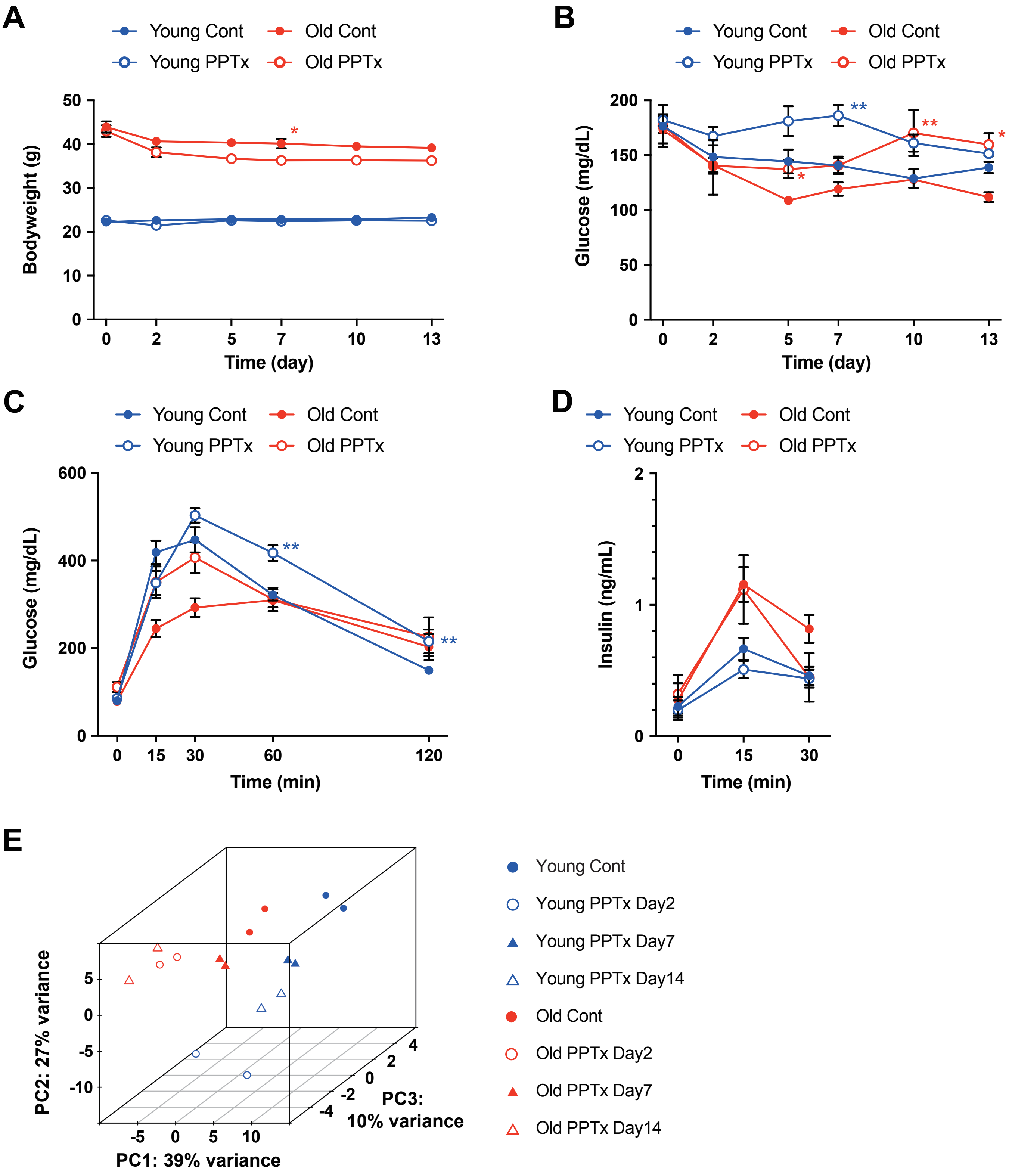

Figure S1. Phenotypical changes of young and old mice by partial pancreatectomy (PPTx).
(A) Changes of body weight. (B) Casual blood glucose levels. (C, D) Oral glucose tolerance test was performed 2 weeks after starting the observation: Levels of glucose (C) and insulin (D) were plotted. Numbers of mice examined were young control n=5, young PPTx n=5, old control n=5 and old PPTx n=4. Values are means±SEM. * and ** indicate $P < 0.05$ and $P < 0.01$ (vs control, Mann-Whitney U test), respectively. (E) PCA plot of bulk RNA-sequencing. Islets were isolated 2, 7 and 14 days after the surgical operation in PPTx mice. Related to Figure 1.

Figure S2

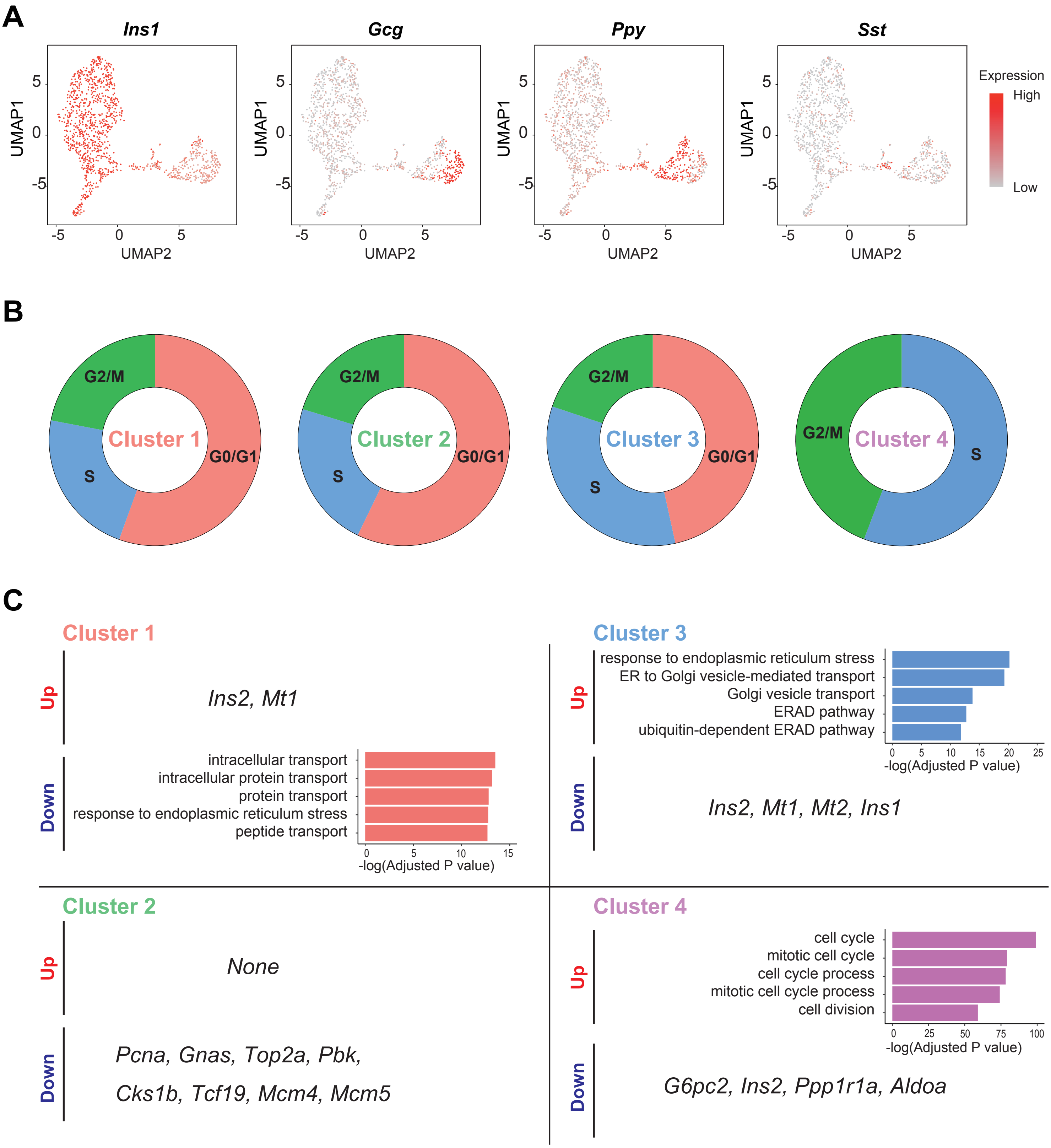

Figure S2. Cell taxonomy and cell cycle state of islet cells in young control and PPTx mice.
(A) Expressions of *Ins1*, *Gcg*, *Ppy* and *Sst* were shown on UMAP plot. (B) CellCycleScoring-defined Composition of G0/G1, S and G2/M cells in each UMAP Cluster. (C) GO terms significantly enriched in up-regulated or down-regulated genes. Gene names were listed when no significant GO term was enriched in their differentially expressed genes. All results were shown in Table S2. Related to Figure 2.

Figure S3

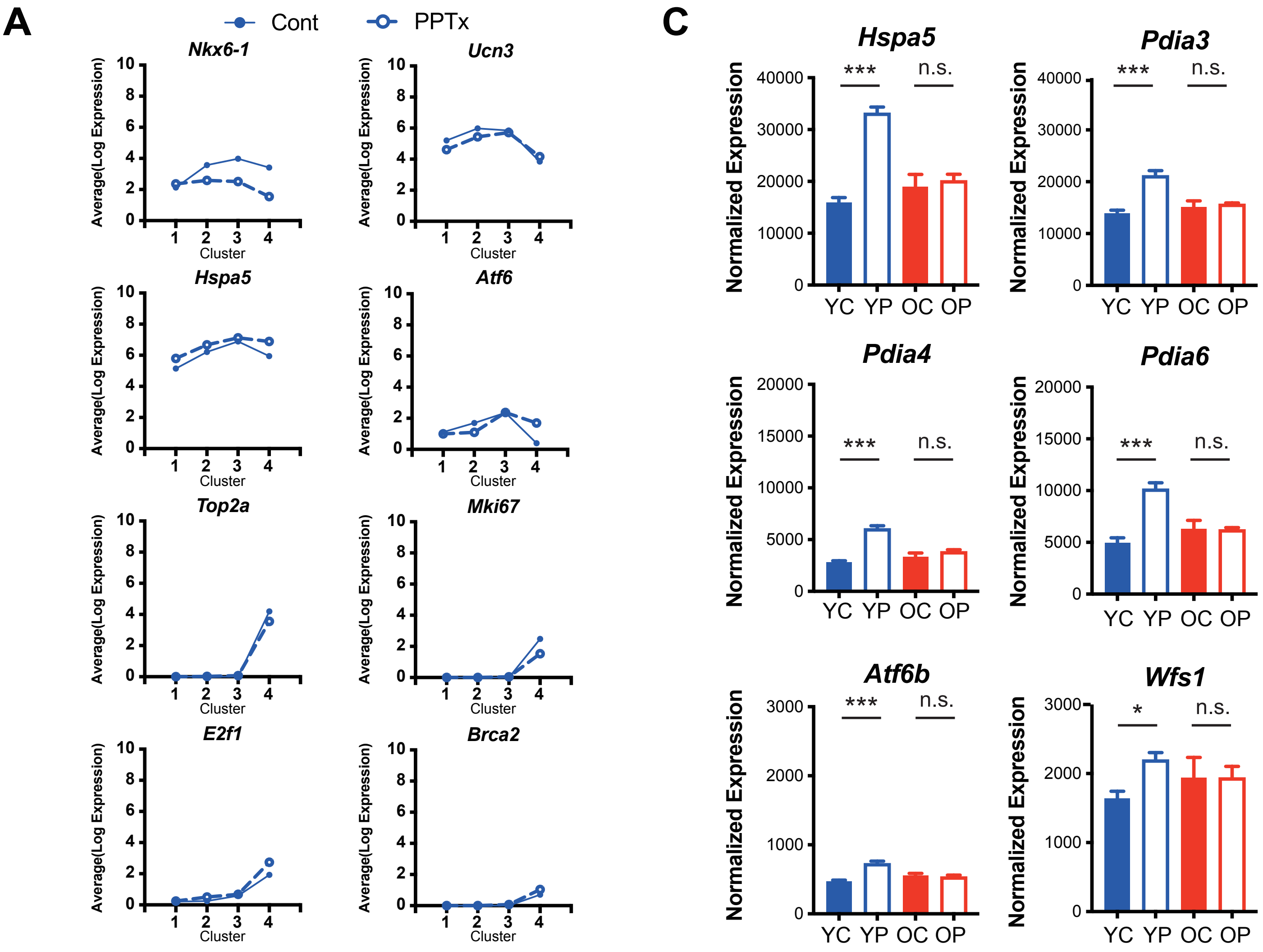

B

Cluster 1 → 2 up		Cluster 2 → 3 up		Cluster 3 → 4 up	
**TF**	**P value**	**TF**	**P value**	**TF**	**P value**
Elk1	1e-89	Elk1	1e-108	NFY	1e-50
ELF1	1e-86	Elk4	1e-107	E2F7	1e-45
Elk4	1e-85	ELF1	1e-100	E2F4	1e-41
ETS	1e-84	ETS	1e-92	E2F1	1e-35
Fli1	1e-61	Fli1	1e-74	Sp1	1e-31
GABPA	1e-58	GABPA	1e-65	KLF1	1e-25
Sp1	1e-57	ETV4	1e-64	E2F3	1e-24
ETV4	1e-53	Sp1	1e-61	E2F	1e-24
Ronin	1e-44	ETV1	1e-51	KLF3	1e-24
GFY-Staf	1e-43	Elf4	1e-44	Sp5	1e-22

Figure S3. A sequential alteration of gene expression in a transition to replication and candidates regulating a gene network.
(A) The alteration of representative gene expressions both in control and PPTx group. Values indicates the average of the logarithm expressions. (B) Upstream motifs of differentially up-regulated genes in cluster 2 compared to cluster 1, in cluster 3 to 2 and in cluster 4 to 3 were predicted using HOMER. Top 10 results were shown. (C) Normalized expression values of ER stress related genes in bulk RNA-seq analysis were shown. YC indicates young control, YP does young PPTx, OC does old control and OP does old PPTx. Values are mean ± SEM. *, $P < 0.05$, ***, $P < 0.001$ (Mann-Whitney U test). Related to Figure 3.

Table S2. CellCycleScoring-defined Composition of G0/G1, S and G2/M cells in each UMAP Cluster of control and PPTx islets. Related to Figures 2D and S2B.

The number of cells assigned to each phase of cell cycle was divided by total number of cells in the Cluster 1-4.

	G1	S	G2M	Total cell number
**Control**				
Cluster 1	63.4%	17.0%	19.6%	112
Cluster 2	69.5%	11.9%	18.6%	177
Cluster 3	65.5%	17.2%	17.2%	58
Cluster 4	0%	23.1%	76.9%	13
**PPTx**				
Cluster 1	54.5%	30.7%	14.8%	88
Cluster 2	50.7%	30.5%	18.8%	213
Cluster 3	43.6%	36.3%	20.1%	204
Cluster 4	0%	64.1%	35.9%	39

TRANSPARENT METHODS

- KEY RESOURCES TABLE
- CONTACT FOR REAGENT AND RESOURCE SHARING
- EXPERIMENTAL MODEL AND SUBJECT DATAILS
    - Animals
- METHOD DETAILS
    - Partial Pancreatectomy
    - Histological analyses
    - Islet isolation and RNA extraction
    - Bulk RNA sequencing analysis (Bulk RNA-seq)
    - Single-cell mRNA sequencing analysis (scRNA-seq)
    - Data analysis
- QUANTIFICATION AND STATISTICAL ANALYSIS
- DATA AVAILABILITY

CONTACT FOR REAGENT AND RESOURCE SHARING

Reagents and resources included in the current study are available from the corresponding author on reasonable request. All requests will be fulfilled by Nobuya Inagaki (inagaki@kuhp.kyoto-u.ac.jp).

KEY RESOURCES TABLE

REAGENT or RESOURCE	SOURCE	IDENTIFIER
**Antibodies**		
Rabbit anti-insulin	Abcam	Cat#ab181547
Rat anti-BrdU	Abcam	Cat#ab6326
**Chemicals, Peptides, and Recombinant Proteins**		
5-Bromo-2′-deoxyuridine (BrdU)	Sigma-Aldrich	Cat# B5002-5G
Trypsin-EDTA	Thermo Fisher Scientific	Cat#15400-054
RPMI 1640 medium	Thermo Fisher Scientific	Cat#31800-022
**Critical Commercial Assays**		
UltraSensitive PLUS Mouse Insulin ELISA	Morinaga	Cat#49170-53
SMARTer Ultra Low Input RNA Kit for Sequencing - v3	Takara Bio Inc.	Cat#634851
Nextera XT DNA Sample Preparation kits	Illumina	Cat#FC-131-1096
SureCell WTA 3′ Library Prep Kit for the ddSEQ System	Illumina	Cat#20014279
Qubit RNA HS Assay Kit	Thermo Fisher Scientific	Cat#Q32855
Agilent RNA 6000 Pico Kit	Agilent Technologies, Inc.	Cat#5067-1513

High Sensitivity D5000 ScreenTape	Agilent Technologies, Inc.	Cat#5067-5592
**Deposited Data**		
Raw data files for RNA sequencing	NCBI Gene Expression	GSE152730 GSE152731
**Experimental Models: Organisms/Strains**		
Mouse: C57BL/6	Japan SLC, Inc.	
**Software and Algorithms**		
R−3.5	R project	https://www.r-project.org
Seurat-3.1	Satija lab	https://satijalab.org/seurat
Monocle-2.4	Trapnell lab	http://cole-trapnell-lab.github.io/monocle-release/
HISAT2-2.0	Pertea et al. 2016.	https://ccb.jhu.edu/software/hisat2/manual.shtml
HTSeq-0.8	Anders, Pyl, and Huber 2015.	https://htseq.readthedocs.io/en/master/
biocManager-1.30	Bioconductor project	https://bioconductor.org
DESeq2-1.22	Love, Huber, and Anders 2014.	https://bioconductor.org/packages/release/bioc/html/DESeq2.html
GOseq-1.34	Young et al. 2010.	https://bioconductor.org/packages/release/bioc/html/goseq.html
Cell Ranger-2.1	10x Genomics	

Ingenuity Pathway Aanalysis (IPA)	QIAGEN	https://digitalinsights.qiagen.com/products-overview/discovery-insights-portfolio/analysis-and-visualization/qiagen-ipa/
HOMER-4.1	Benner Lab	http://homer.ucsd.edu/homer/motif/
iReguron-1.3	Stein Aerts Lab	http://iregulon.aertslab.org
GraphPad PRISM 8	GraphPad Software	https://www.graphpad.com/scientific-software/prism/
BZ Analyzer	KEYENCE	

EXPERIMENTAL MODEL DETAILS AND SUBJECT DATAILS

Animals

C57BL/6J male mice were purchased from SLC Japan, Inc. (Hamamatsu, Japan). Animals were housed in a 14h light-10h dark cycle with free access to water and standard chow. Experiments on animals were approved by the Animal Research Committee of Kyoto University Graduate School of Medicine (MedKyo18249) and all experiments were performed in accordance with relevant guidelines and regulations of the Animal Research Committee of Kyoto University Graduate School of Medicine.

METHOD DETAILS

Partial pancreatectomy (PPTx)

PPTx was performed on 8-week-old and 52-week-old male C57BL/6J mice as described previously (Peshavaria et al., 2006). Mice were anesthetized with isoflurane inhalation, and a midline abdominal incision was made. The splenic side of the pancreas was excised partially via the line between the pylorus ring and the small intestine, after breaking mesenteric connections from the pancreas to the greater curvature of the stomach, the pylorus ring, the colon and the retroperitoneum. The weight of the resected pancreas was approximately 50% of that of total pancreas estimated by those of mice of the same age and similar bodyweight. Mice at the same age without any operation were examined as control. Body weight and casual blood glucose via tail vein using Glutest Neo Sensor (Sanwa Kagaku Kenkyusho, Japan) were examined before and until 13 days after the operation. Oral glucose tolerance test was performed at 9 a.m. on day 14 after fasting mice from 5 p.m. on the previous day. Mice were dosed orally with 2g/kg glucose, and blood samples were collected at various time points using heparinized calibrated glass capillary tubes (Catalogue No. 2-000-044-H; Drummond Scientific Company). Blood glucose levels were determined using Glutest Neo Sensor. Plasma samples were prepared by centrifugation of the blood samples at 9,000xg for 10min, and were subjected to insulin measurement using UltraSensitive PLUS Mouse Insulin ELISA kit (Catalogue No. 49170-53; Morinaga, Tokyo, Japan).

Histological analyses

The β-cell replication rate was estimated immunohistochemically by 5-bromo-2′-deoxyuridine (BrdU) labeling as previously described with minor modifications (El Ouaamari et al. 2013). Water containing 1mg/mL BrdU (Catalogue No. B5002; Merck KGaA, Darmstadt, Germany) was supplied for 4 days before the dissection or intraperitoneal injection of 100 mg/kg BrdU was done 6 hours before the dissection. The resected pancreatic tissues were fixed with 4% paraformaldehyde and embedded in paraffin. After deparaffinization, antigen-retrieval by heating in 10mM citrate buffer (pH 6.0) and blocking by blocking buffer (PBS with 10% Goat Serum and 0.2% Triton X-100), five slices (more than 120 μm apart) of each pancreas were incubated at room temperature overnight with primary antibodies [i.e., rabbit anti-insulin antibody (Catalogue No. ab181547; Abcam, USA; 200-fold dilution in blocking buffer) and rat anti-BrdU antibody (Catalogue No. ab6326; Abcam, USA; 100-fold dilution in blocking buffer)] and then for 1hr at room temperature with secondary antibodies [i.e., Alexa Fluor 488-conjugated goat anti-rabbit IgG (H+L) antibody (Catalogue No. A-11034; Thermo Fisher Scientific, USA; 200-fold dilution in blocking buffer) and Alexa Fluor 546 goat anti-rat antibody (Catalogue No. A-11081; Thermo Fisher Scientific, USA; 200-fold dilution in blocking buffer)]. The samples were washed 4 times with PBS and then incubated with PBS containing 4',6-diamidino-2-phenylindole (DAPI) at the final concentration of 0.01 mg/mL for 15 min at room temperature. The samples were then washed 4 times with PBS and mounted using VECTASHIELD Mounting Medium (Catalogue No. H-1000; VECTOR LABORATORIES, INC., USA). Each slide was observed using BZ-X700 (KEYENCE, Japan) through filters (470/40 -DM 495- 525/50: FITC), (545/25 -DM 565- 605/70: mTIRC) and (360/40 -DM 400- 460/50: DAPI). More than 10 islets per slide (>50 slides per one mouse) were analyzed; the ratio of BrdU positive cell number to cell number estimated by DAPI in pancreatic islets was calculated by BZ Analyzer (KEYENCE, Japan).

Islet isolation and RNA extraction

Islets of Langerhans were isolated by the collagenase digestion technique (Sutton et al., 1986; Usui et al., 2019). Briefly, Hanks' balanced salt solution (HBSS) (Catalogue No. 05905; Nissui Pharmaceutical Co., Ltd. Tokyo, Japan) containing 5 mM $NaHCO_3$ and 0.5 mg/ml (weight/volume)

collagenase P (Catalogue number 11213865001, Roche) was injected to the mouse pancreas via the bile duct. Subsequently, the pancreas was removed and further digested in a 50 mL tube in a shaking water bath at 37℃ for 30 min. The digested pancreas was washed 2 times by ice-cold Krebs–Ringer bicarbonate (KRB) buffer [129.4 mM NaCl, 5.2 mM KCl, 2.7 mM CaCl2, 1.3 mM KH_2PO_4, 1.3 mM $MgSO_4$, and 24.8 mM $NaHCO_3$ (pH 7.4)] equilibrated with 5% CO_2/95% O_2 and containing 2.8 mM glucose, and then suspended in 4 ml of histopaque 1119 (Catalogue number 11191, Sigma) and transferred to a clean glass tube. Two ml of histopaque 1077 (Catalogue number 10771, Sigma) and 2 ml of histopaque 1050 (prepared by mixing two volumes of histopaque 1077 with one volume of distilled water) were then sequentially overlaid to perform density gradient separation. After 800×g centrifugation for 10 min at room temperature, the islets found in the inter-phase between histopaque 1050 and histopaque 1077 were collected and washed 2 times in ice-cold KRB buffer. The resulting islets were transferred to a large dish filled with approximately 50 ml of ice-cold KRB buffer and hand-picked into a 1.5-ml tube before total RNA preparation. After 200×g centrifugation for 1 min at 4℃ and removal of supernatant, a tissue pellet was made and frozen in liquid nitrogen with RNAlater Stabilization Solution (Catalogue number 7020, Thermo Fisher Scientific).

Bulk RNA sequencing (Bulk RNA-seq)

Total RNA was prepared from the frozen pellets of isolated islets using RNeasy mini kit following the manufacture's instruction (Catalogue number 74104, Qiagen); the RNA concentration was measured using the Qubit RNA HS Assay Kit (Catalogue number Q32855, Thermo Fisher Scientific). Quality of total RNA was assessed by Agilent 2100 Bioanalyzer System (Agilent Technologies, Inc., Santa Clara, CA, USA) with Agilent RNA 6000 Pico Kit (catalogue number 5067-1513, Agilent Technologies, Inc.), and RNA samples with RNA Integrity Number (RIN) value more than 7.5 were subjected to bulk RNA-seq. Bulk RNA-seq of whole islets was performed using 10 ng of total RNA from isolated islets. Each sample of islets were collected from 1-2 mice. Total RNA was reverse transcribed with SMARTer Ultra Low Input RNA Kit for Sequencing - v3 (catalogue number 634851, Takara Bio Inc.), and libraries were prepared with Nextera XT DNA Sample Preparation kits (Catalogue number FC-131-1096, Illumina). The libraries were sequenced by Hiseq2500 following the manufacturer's instruction. Single read 93-8-8bp were mapped to the UCSC mouse transcriptome reference (mm9) by HISAT2 (version 2.0.5) (Pertea et al., 2016) and uniquely aligned reads within Ensembl gene annotations were used to quantify gene expressions as counts using HTSeq (version 0.8.0) (Anders et al., 2015). For principal component analysis, islets of control, 2, 7 and 14 days after PPTx both in young and old mice were sequenced and normalized by DESeq2 (1.22.2) (Love et al., 2014) (n=2, each group). Differentially expressed analyses were conducted between islets of control and 2 days after PPTx both in young and old mice using DESeq2 (1.22.2) and the genes of adjusted P values < 0.05 were defined as differentially expressed genes (DEGs) (n=4, each group). Following Gene Ontology analysis of obtained DEGs was performed using GOseq (version 1.34.1) (Young et al., 2010).

Single-cell mRNA sequencing (scRNA-seq)

More than 120 isolated islets from 1 to 3 mice per sample were collected into 0.5mL tubes in RPMI 1640 medium (Catalogue No. 31800-022; Thermo Fisher Scientific, USA) containing 10% fetal bovine serum (FBS), 10 mM HEPES, 5 mM $NaHCO_3$, 1 mM sodium pyruvate, 100 U/ml penicillin, 100 mg/ml streptomycin and 11.1 mM glucose on ice. Four samples as control and four samples as PPTx group were prepared. After 200×g-centififugation for 1 min at 4℃, the collected islets were washed once in 500 µL of calcium/magnesium free (CMF) buffer (136.9 mM NaCl, 4.0 mM KCl, 11.9 mM $NaHCO_3$, 11.1 mM glucose, and 0.42 mM NaH_2PO_4, 0.18 mM KH_2PO_4). The islets were then resuspended in 200 µL of CMF containing 2 µL of 0.5% (wt/vol) Trypsin-EDTA (Catalogue number 15400-054, Thermo Fisher Scientific) (final concentration, 0.005%), incubated for 3 min at 37℃ in a water bath, and dispersed by pipetting 50 times using a Gilson P200 pipet and 200 µl-pipet tips (Catalogue number 7231, WakenBtech Co., Ltd.). Cells were collected at the bottom of 0.5 mL tubes by 1000×g centrifugation for 5 min at 4℃ and resuspended in CMF before being applied. Cell numbers were counted by TC20 Automated Cell Counter (Bio-Rad Laboraroties, Inc.) and observed with a polarized light microscope. Single cell isolation was performed by applying dissociated islet cells to a ddSEQ Single-Cell Isolator (Bio-Rad Laboratories, Inc., USA) as described previously (Romagnoli et al., 2018). Libraries were prepared by SureCell WTA 3′

Library Prep Kit for the ddSEQ System (Catalogue number 20014279, Illumina, Inc., USA) using approximately 12,500 cells in single-cell resuspension as described previously (Tran et al., 2019). Single-cell droplets encapsulated in oil with Barcode Suspension Mix and Cell Suspension Mix were brought to reverse transcription. Second strand synthesis was conducted using the library of first strand cDNA. Quality of amplified library was assessed by Agilent 2200 TapeStation (Agilent Technologies, Inc.) with High Sensitivity D5000 ScreenTape (Catalogue number 5067-5592, Agilent Technologies, Inc.). DNA were tagged with DNA adaptors and amplified, followed by sequencing on Hiseq2500 as the manufacturer's instructions. FASTQ files were made using bcl2fastq v2.20.0.422. Paired end 68-8-0-75 bp were aligned to the mouse reference genome mm10, followed by filtering, debarcoding and UMI counting using the Cell Ranger v2.1.0 pipeline (10X Genomics).

Data analysis

scRNA-seq data analysis was conducted using R version 3.5.1 and Seurat Version 3.1(Butler et al., 2018; Stuart et al., 2019). Cells were removed if the number of detected genes was less than 100 or more than 3000, or read count was less than 100 or more than 20000. Genes were also removed if they were expressed in less than 3 cells. Normalization, scaling, dimensional reduction and clustering by PCA and UMAP were processed on Seurat pipeline as follows. After removing unwanted cells, gene expressions were normalized by for each cell by the total expression, multiplied by 10,000 and their natural logarithm were taken. Top 2000 highly variable genes calculated by FindVariableFeatures function with vst selection, were used for following PCA analysis using RunPCA function. The distance between cells were calculated using FindNeighbors function with dimension 1 to 7. Clustering was performed using FindClusters function with 0.5 as the resolution. Finally, clustered cells were plotted on UMAP space using RunUMAP function. For cell taxonomy, the expressions of *Ins1*, *Gcg*, *Ppy* and *Sst* were used as previously described (Butler et al., 2018). Cluster specific genes were calculated by FindAllMarkers function using Wilcoxon rank sum test in Seurat package and adjusted *P* value < 0.05 was defined as significant. GO analysis for cluster specific genes were conducted using goseq. Cell cycle analysis was conducted using CellCycleScoring function with a dataset in a previous report as guided on developers' website (Nestorowa et al., 2016).

Pseudo-time analysis was conducted using Monocle version 2.4.0 (Qiu et al., 2017; Trapnell et al., 2014). Cells of β-cell populations (i.e., Cluster 1-4) and filtered genes on UMAP analysis (i.e., expressed in at least 3 cells) were used. Pseudo-time of each cell was calculated by reduceDimension function by DDRTree method. Genes for ordering were selected by q value less than 0.05 by differentialGeneTest function following to reduceDimension and clusterCells without marker genes as unsupervised manner. After plotting the trajectory along with pseudo-time, cells from cluster 1 to 4 were selected and the scaled expression of all genes were drawn using plot pseudo-time heatmap function. All genes were classified into 7 groups depending on their expression patterns. GO terms enriched in genes of each cluster were calculated by GOseq. Dynamic expression of cell cycle-specific *cyclins* was shown as Z score of mean expression in each cluster.

Pathway analysis was conducted using Ingenuity Pathway Analysis (IPA, Qiagen) for differentially expressed genes between cluster 1 and 2, 2 and 3 or 3 and 4 with *P* value calculated by Wilcoxon rank sum test and fold change of mean expression. Transcription factor binding motif analysis was conducted using HOMER software version 4.11(Heinz et al., 2010). Motifs were searched against DNA sequences from -300 to +50 around the transcription start sites of the differentially expressed genes between β-cell clusters. Upstream analysis for genes devided into 7 groups in pseudo-time heatmap was conducted using iReguron (Janky et al., 2014). The candidate upstream transcription factors which were also included in the group of the same expression pattern (i.e., decreased genes, groups 1-3; transiently up-regulated genes, group 4, 5; elevated genes, group 6-7) were listed.

Comparison of cluster4 genes with human datasets were performed using previously published dataset (Ackeifi et al., 2020; Arda et al., 2016; Wang et al., 2017). As for insulinoma dataset by Wang and juvenile human islet dataset by Arda, differentially expressed genes described in their reports were used. Genes upregulated in human islets treated by harmine were calculated by more than 2-fold elevation from expression values in the paper.

QUANTIFICATION AND STATISTICAL ANALYSIS.

Data of mouse phenotypes, immunohistochemical examinations and the proportion of each cluster in cell number of scRNA-seq (Figures 1C, 2C, S1A-S1D and S3C) are expressed as mean ± standard error of the mean (SEM). Comparison between two groups was performed by the Mann-Whitney U-test. The statistical analysis was carried out using GraphPad Prism software Version 8.0.2 (San Diego, CA, USA).

DATA AVAILABILITY

The accession number of NCBI Gene Expression Omnibus for scRNA-seq and bulk RNA-seq data reported in this manuscript is GSE152730 and GSE152731, respectively.

SUPPREMENTAL REFERENCES

Ackeifi, C., Swartz, E., Kumar, K., Liu, H., Chalada, S., Karakose, E., Scott, D.K., Garcia-Ocana, A., Sanchez, R., DeVita, R.J., et al. (2020). Pharmacologic and genetic approaches define human pancreatic beta cell mitogenic targets of DYRK1A inhibitors. JCI Insight *5*.

Anders, S., Pyl, P.T., and Huber, W. (2015). HTSeq--a Python framework to work with high-throughput sequencing data. Bioinformatics *31*, 166-169.

Arda, H.E., Li, L., Tsai, J., Torre, E.A., Rosli, Y., Peiris, H., Spitale, R.C., Dai, C., Gu, X., Qu, K., et al. (2016). Age-Dependent Pancreatic Gene Regulation Reveals Mechanisms Governing Human beta Cell Function. Cell Metab *23*, 909-920.

Butler, A., Hoffman, P., Smibert, P., Papalexi, E., and Satija, R. (2018). Integrating single-cell transcriptomic data across different conditions, technologies, and species. Nat Biotechnol *36*, 411-420.

Heinz, S., Benner, C., Spann, N., Bertolino, E., Lin, Y.C., Laslo, P., Cheng, J.X., Murre, C., Singh, H., and Glass, C.K. (2010). Simple combinations of lineage-determining transcription factors prime cis-regulatory elements required for macrophage and B cell identities. Mol Cell *38*, 576-589.

Janky, R., Verfaillie, A., Imrichova, H., Van de Sande, B., Standaert, L., Christiaens, V., Hulselmans, G., Herten, K., Naval Sanchez, M., Potier, D., et al. (2014). iRegulon: from a gene list to a gene regulatory network using large motif and track collections. PLoS Comput Biol *10*, e1003731.

Love, M.I., Huber, W., and Anders, S. (2014). Moderated estimation of fold change and dispersion for RNA-seq data with DESeq2. Genome Biol *15*, 550.

Nestorowa, S., Hamey, F.K., Pijuan Sala, B., Diamanti, E., Shepherd, M., Laurenti, E., Wilson, N.K., Kent, D.G., and Gottgens, B. (2016). A single-cell resolution map of mouse hematopoietic stem and progenitor cell differentiation. Blood *128*, e20-31.

Pertea, M., Kim, D., Pertea, G.M., Leek, J.T., and Salzberg, S.L. (2016). Transcript-level expression analysis of RNA-seq experiments with HISAT, StringTie and Ballgown. Nat Protoc *11*, 1650-1667.

Peshavaria, M., Larmie, B.L., Lausier, J., Satish, B., Habibovic, A., Roskens, V., Larock, K., Everill, B., Leahy, J.L., and Jetton, T.L. (2006). Regulation of pancreatic beta-cell regeneration in the normoglycemic 60% partial-pancreatectomy mouse. Diabetes *55*, 3289-3298.

Qiu, X., Mao, Q., Tang, Y., Wang, L., Chawla, R., Pliner, H.A., and Trapnell, C. (2017). Reversed graph embedding resolves complex single-cell trajectories. Nat Methods *14*, 979-982.

Romagnoli, D., Boccalini, G., Bonechi, M., Biagioni, C., Fassan, P., Bertorelli, R., Sanctis, V., Leo, A.D., Migliaccio, I., Malorni, L., et al. (2018). ddSeeker: a tool for processing Bio-Rad ddSEQ single cell RNA-seq data. BMC Genomics *19*, 960.

Stuart, T., Butler, A., Hoffman, P., Hafemeister, C., Papalexi, E., Mauck, W.M., 3rd, Hao, Y., Stoeckius, M., Smibert, P., and Satija, R. (2019). Comprehensive Integration of Single-Cell Data. Cell *177*, 1888-1902 e1821.

Sutton, R., Peters, M., McShane, P., Gray, D.W., and Morris, P.J. (1986). Isolation of rat pancreatic islets by ductal injection of collagenase. Transplantation *42*, 689-691.

Tran, K.A., Pietrzak, S.J., Zaidan, N.Z., Siahpirani, A.F., McCalla, S.G., Zhou, A.S., Iyer, G., Roy, S., and Sridharan, R. (2019). Defining Reprogramming Checkpoints from Single-Cell Analyses of Induced Pluripotency. Cell Rep *27*, 1726-1741 e1725.

Trapnell, C., Cacchiarelli, D., Grimsby, J., Pokharel, P., Li, S., Morse, M., Lennon, N.J., Livak, K.J., Mikkelsen, T.S., and Rinn, J.L. (2014). The dynamics and regulators of cell fate decisions are revealed by pseudotemporal ordering of single cells. Nat Biotechnol *32*, 381-386.

Usui, R., Yabe, D., Fauzi, M., Goto, H., Botagarova, A., Tokumoto, S., Tatsuoka, H., Tahara, Y., Kobayashi, S., Manabe, T., et al. (2019). GPR40 activation initiates store-operated Ca(2+) entry and potentiates insulin secretion via the IP3R1/STIM1/Orai1 pathway in pancreatic beta-cells. Sci Rep *9*, 15562.

Wang, H., Bender, A., Wang, P., Karakose, E., Inabnet, W.B., Libutti, S.K., Arnold, A., Lambertini, L., Stang, M., Chen, H., et al. (2017). Insights into beta cell regeneration for diabetes via integration of molecular landscapes in human insulinomas. Nat Commun *8*, 767.

Young, M.D., Wakefield, M.J., Smyth, G.K., and Oshlack, A. (2010). Gene ontology analysis for RNA-seq: accounting for selection bias. Genome Biol *11*, R14.
